# Sunitinib Induced Immune Thrombocytopenia

**Published:** 2015

**Authors:** Ramin Shekarriz, Neda Koulaeinejad, Anahita Nosrati, Ebrahim Salehifa

**Affiliations:** a *Department of Hematology-Oncology, Gastrointestinal Cancer Research Center, Imam Khomeini Hospital, Mazandaran University of Medical Sciences, Sari, Iran. *; b *Faculty of Pharmacy, Mazandaran University of Medical Sciences, Sari, Iran.*; c *Department of Pathology, Imam Khomeini Hospital, Mazandaran University of Medical Sciences, Sari, Iran. *; d *Department of Clinical Pharmacy, Pharmaceutical Research Center, Mazandaran University of Medical Sciences, Sari, Iran.*

**Keywords:** Sunitinib, Carcinoma, Thrombocytopenia, Tyrosine kinase

## Abstract

Sunitinib is an oral tyrosine kinase inhibitor which prevents tumor growth and metastatic progression. It was approved for treatment of advanced renal cell cancer, gastrointestinal stromal tumor and advanced pancreatic neuroendocrine tumors. It has several adverse reactions on multi organ systems including hematologic system. Although the neutropenia and thrombocytopenia commonly happens as Grade 3 or 4 abnormalities following bone marrow suppression, in the rare cases, the immune mediated abnormality may drive the sunitinib-induced hematologic disorder. In this report, we present a case of immune-mediated thrombocytopenia induced by sunitinib. One month after first treatment cycle with sunitinib, leucopenia and thrombocytopenia were occurred. The patient had a normal bone marrow aspiration and biopsy, the thrombocytopenia was resistant to platelet transfusion which successfully was treated with prednisolone.

## Introduction

Sunitinib is an oral tyrosine kinase receptor inhibitor approved for advanced renal cell cancer, gastrointestinal stromal tumor and advanced pancreatic neuroendocrine tumors. Several receptors are inhibited with this drug which is included vascular endothelial growth factor receptors (VEGFR1, 2, 3), platelet-derived growth factor receptors (PDGFR), stem cell factor receptor (KIT), Fms-like tyrosine kinase-3 (FLT3), colony-stimulating factor receptor type 1 (CSF-1R), and the glial cell-line derived neurotrophic factor receptor (RET). Thus sunitinib prevents tumor growth and metastatic progression and was approved by US Food and Drug Administration (FDA) in 2006 for the treatment of metastatic renal cell carcinoma (mRCC) and gastrointestinal stromal tumor (GIST). Sunitinib has multiple adverse effects on body organ systems such as hematologic system. Neutropenia and thrombocytopenia commonly happened as Grade 3 or 4 abnormalities (1, 2).

In this report, we will present a rare case of sunitinib-induced thrombocytopenia that was related to immune system abnormality. To the best of our knowledge, a few case reports have been presented in the literature about sunitinib induced immune thrombocytopenia (3-5).

*Case Presentation*

A 64 years old woman with complains of general weakness and malaise, anemia (Hb: 8 gr/dl) and elevated inflammatory markers (erythrocyte sedimentation rate and C-reactive protein, 115 mm/h and 120 mg/l, respectively) was referred to a University affiliated subspecialty clinic in Sari, Mazandaran, Iran. In Ultrasound evaluation, a mass in left kidney was detected and confirmed by CT scan with radiologic picture of renal cell carcinoma (RCC). There was no evidence of metastasis in bone, lungs, abdomen and pelvis. Nephrectomy was performed and pathological evaluation reported an intermediate grade RCC without capsule, vascular and peripheral invasion but distal margin of ureter was invaded. After nephrectomy, patient was not referred to oncologist. She came back with malaise and feeling of heaviness in the left side of abdomen after two years. In the blood tests, sharp increase of ESR and recurrence of anemia (Hb: 10 g/dl) was found. In the CT scan, large mass (80 × 70 mm) at the site of previous surgery and metastatic nodules in both lungs were discovered. 50 mg sunitinib (Sutent^®^; Pfizer Pharmaceuticals, New York) was administered daily for 4 weeks followed by 2 weeks off. One month after first treatment cycle, leucopenia and thrombocytopenia were occurred on CBC control tests (WBC: 2,300 /mm^3^, PLT: 20, 000/µl).

**Figure 1:**
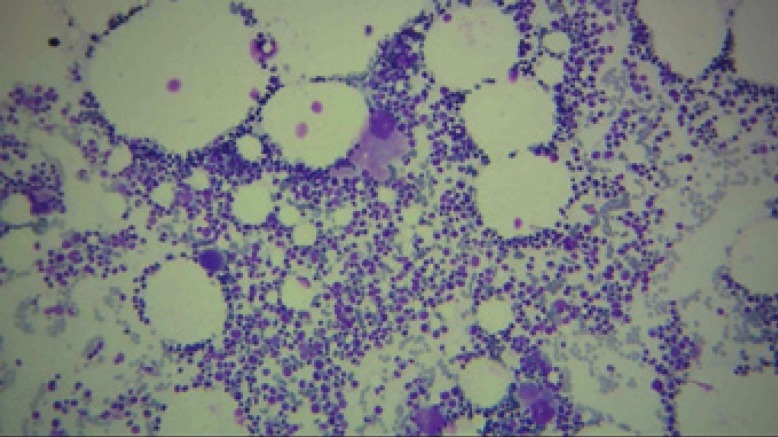
The bone marrow aspiration sample (*10) demonstrating the sufficient cellularity and megakaryocytes

**Figure 2:**
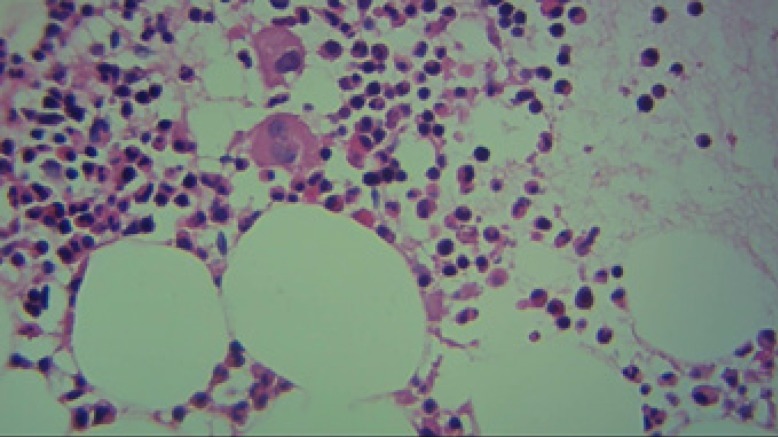
The bone marrow biopsy sample (*40) demonstrating partially normocellularity and presence of megakaryocytes

**Figure 3:**
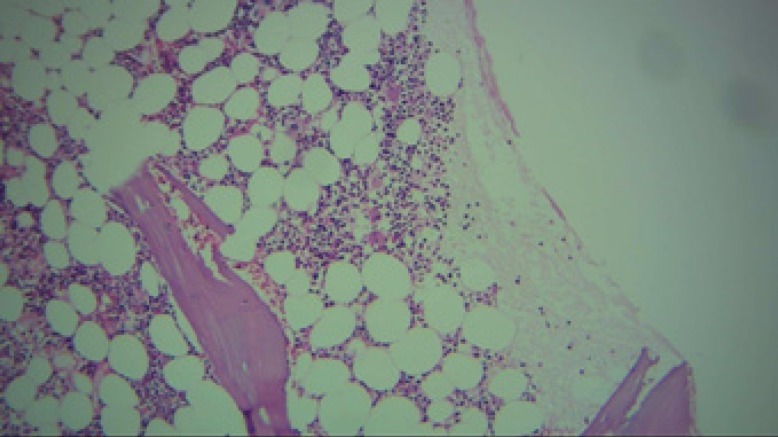
The bone marrow biopsy sample (*4) demonstrating sufficient cellularity and megakaryopoiesis

Regarded to severe thrombocytopenia and ecchymosis, the patient hospitalized. At first, due to a platelet count of 15000/µl and ecchymosis, she was received 5 units of platelet. After 6 h, the platelet count raised to 40000/µl followed by a platelet count of 20000/µl on next day. Despite of platelet transfusion, thrombocytopenia was continued. Bone marrow aspiration and biopsy showed no metastasis. Unexpectedly, cellularity and number of megakaryocyte with regard to age and gender was normal (Figures 1 to 3).

In addition to supportive therapy, because of prolongation of thrombocytopenia (less than 30,000/µl for 10 days) and observation of normal megakaryocyte, low dose prednisolone (15 mg daily) was started. Within 4 weeks of discontinuation of sunitinib, platelet count began to rise and eventually reached to 200,000/µl. Then Sorafenib was administered for two treatment cycles with no recurrent thrombocytopenia. Patient discontinued treatment due to financial problems and was treated with supportive therapy including analgesia, blood transfusion and TPN (total parenteral nutrition). She died one year later, while been on supportive therapy.

## Discussion

Immune thrombocytopenia (ITP) is mediated by humoral and cellular immunity. It could be secondary to other diseases. For example infections, other autoimmune or immunodeficiency disorders, malignancy, liver and bone marrow disease, recent vaccination and transfusions and inherited thrombocytopenia syndromes (6). Drug induced immune thrombocytopenia (DITP) may occur with chemotherapy and immunosuppressive agents (7). According to previous medical literature, ITP has been occurred in a patient with RCC (8). So other causes of thrombocytopenia must be ruled out to verify the diagnosis of DITP. In our case, the patient did not use immunosuppressive medications and there is no sign of infection and other autoimmune and hepatic disease. We evaluate validity of this report by Naranjo nomogram that it acquired a score of 5 (probable adverse reaction) (9). Although measuring the drug dependent antibodies (DDAb) is a more reliable method for evaluation of the causality relationship (7), it was not available for us. Despite of this limitation, considering the normal biopsy of bone marrow we believe that ITP can explain the cause of acute thrombocytopenia in our case. In addition, ITP appeared after the drug was administered and resolved after the suspected drug was withdrawn and the patient responded to treatment with corticosteroid.

Sunitinib has been documented causing myelosuppression resulting thrombocytopenia in literatures. Motzer *et al.* reported grade 3 of thrombocytopenia in 8 of 65 (12%) patients whom experience all grades (10). In other study, in 78 patients, 18 (23%) and 4 (5%) were faced with grade 3 and 4, respectively (11). Trinkaus *et al*. reported the first case of ITP secondary to sunitinib and their patient’s platelet count returned to normal after 2 weeks treatment with IVIG (1 g/kg over 2 days), intravenous tranexamic acid and withholding the sunitinib (3). The mentioned treatment was also used successfully by Mutahir *et al*. in the same duration (4). At last, Ansari and George normalized the patient's platelet count by IVIG (0.4 gr/kg/day) and prednisolone (50 mg/day) after 3 weeks (5). Our case was treated by prednisolone (15 mg/day) only and platelet count improved within 4 weeks.

## Conclusion

Sunitinib may induce thrombocytopenia by immune related mechanism. This type of thrombocytopenia is resistant to platelet infusion and could be managed with corticosteroids alone.
